# Point of care SARS-CoV-2 nucleic acid testing in schools improves school attendance

**DOI:** 10.12688/wellcomeopenres.17213.1

**Published:** 2022-01-10

**Authors:** Dami A. Collier, Rachel Bousfield, Effrossyni Gkrania-Klotsas, Ravindra K. Gupta

**Affiliations:** 1 Cambridge Institute of Therapeutic Immunology & Infectious Disease (CITIID), Cambridge, CB2 0SP, UK; 2 Department of Medicine, University of Cambridge, Cambridge, CB2 0QQ, UK; 3 Division of Infection and Immunity, University College London, London, WC1E 6BT, UK; 4 Department of Infectious Diseases, Cambridge University Hospitals, NHS Trust, Cambridge, CB2 0QQ, UK; 5 MRC Epidemiology Unit, University of Cambridge, Cambridge, CB22 0SL, UK; 6 Africa Health Research Institute, Durban, South Africa

**Keywords:** SARS-CoV-2, school, testing, rapid, point of care

## Abstract

**Background:** National lockdowns have led to significant interruption to children’s education globally. In the Autumn term in 2020, school absence in England and Wales was almost five times higher than the same period in 2019. Transmission of SARS-CoV-2 in schools and ongoing interruption to education remains a concern. However, evaluation of rapid point of care (POC) polymerase chain reaction (PCR) testing in British schools has not been undertaken.

**Methods: **This is a survey of secondary schools in England that implemented PCR-based rapid POC testing. The study aims to measure the prevalence of SARS-CoV-2 infection in schools, to assess the impact of this testing on school attendance and closures, and to describe schools experiences with testing. All schools utilised the SAMBA II SARS-CoV-2 testing platform.

**Results:** 12 fee-paying secondary schools in England were included. Between September 1 ^st^ 2020 and December 16 ^th^ 2020, 697 on site rapid POC PCR tests were performed and 6.7% of these were positive for SARS-CoV-2 infection. There were five outbreaks in three schools during this time which were contained. Seven groups of close contacts within the school known as bubbles had to quarantine but there were no school closures. 84% of those tested were absent from school for less than one day whilst awaiting their test result. This potentially saved between 1047 and 1570 days off school in those testing negative compared to the NHS PCR laboratory test. Schools reported a positive impact of having a rapid testing platform as it allowed them to function as fully as possible during this pandemic.

**Conclusions:** Rapid POC PCR testing platforms should be widely available and utilised in school settings. Reliable positive tests will prevent outbreaks and uncontrolled spread of infection within school settings. Reliable negative test results will reassure students, parents and staff and prevent disruption to education.

## Summary

Testing could reduce SARS-CoV-2 transmission in schools and mitigate against school closures and COVID-19 related absences. We evaluated the impact of rapid point of care PCR testing for SARS-COV-2 in schools and found it identified cases promptly and reduced school absence in non-cases.

## Introduction

The COVID-19 pandemic has had an indelible impact on the education of children in the United Kingdom ^1^. National lockdowns have led to significant interruption to children’s education ^2^. Over the Autumn term in the 2020–2021 academic year, the average weekly absence from state-funded primary and secondary schools in England was 13% and this peaked at 28% in the last week of the Autumn term in December 2020 ( school attendance during SARS-CoV-2 outbreak). This compared with an overall absence rate of 4.9% in Autumn 2019 before the COVID-19 pandemic ^3^. Attendance at state-funded secondary schools in 151 local authorities in England fell from 88% in the week of the 10 ^th^ of September 2020 to 68% by the week of the 11 ^th^ of December 2020 ( English secondary school attendance). Further interruptions must be avoided.

The availability of rapid point of care (POC) diagnostic testing has been shown to facilitate timely diagnoses in hospitals ^4– 6^. However, less impressive results have been reported in community settings using antigen tests with lower sensitivity ^7, 8^. Rapid, sensitive and specific SARS-CoV-2 POC testing could help to avoid school absence and school closures.

## Methods

We conducted a survey of SARS-CoV-2 infection in secondary schools in England that had implemented PCR-based rapid POC testing for use in diagnosis and isolation of pupils and staff with SARS-CoV-2 infection on their school premises. All participating schools were fee-paying schools and boarding or mixed boarding/day schools. All schools utilised the SAMBA II SARS-CoV-2 testing platform ^9^ and tests were performed by a school nurse who had received training from the manufacturer.

Schools were identified by convenience sampling either through the Independent Schools’ Bursars Association, who cascaded the invite to join the study to the headteachers of their member schools or through our working knowledge of schools that had purchased their own testing platform. The headteacher of the schools were sent an electronic letter inviting their school to participate. The letter included the participant information and consent forms. Informed consent was obtained from the headteacher in an electronic format. Following this, the school was enrolled in the study. A survey was sent out to the school electronically which was completed by the school nurse and returned weekly. Fully anonymised risk factor data were gathered along with the number and results of SARS-CoV-2 tests carried out in each school (see Extended data 1) ^10^. Data collected includes test performed from the 1 ^st^ of September 2020 and will be collected up till the end of the academic year in August 2021. This initial report is based on data collected in the first term of the academic year up until the 16 ^th^ of December 2020.

Data on new daily cases of COVID-19 stratified by age from South East, South West and East of England were downloaded from the Public Health England website. Data on state-funded school attendance during the COVID-19 was obtained from routinely published Department for Education data on attendance in education and early years settings during the COVID-19 outbreak ( school attendance in England).

The main outcome is the proportional prevalence of SARS-CoV-2 infection at specific time points in the 2020/2021 academic year amongst those tested. Secondary outcomes include the impact of rapid POC SARS-CoV-2 testing on school attendance, school closure and closure of groups of close contacts within the school known as bubbles, which may be a year group, class, house or dorm. Qualitative accounts of participating school’s experiences were collected as an open-ended survey question. These are presented as vignettes, in order to describe more fully the impact of SARS-CoV-2 rapid POC PCR testing in schools. Given that the choice of who to test followed the discretion of each school and may have differed at each school, details of implementing these tests and why students or staff were tested were collected.

Descriptive analyses of demographic and clinical data are presented as median and interquartile range (IQR) when continuous and as frequency and proportion (%) when categorical. The differences in continuous and categorical data were tested using the Mann-Whitney test and Chi-square test, respectively. Logistic regression was used to explore the association between a positive rapid POC result and *a priori* determined risk factors for SARS-CoV-2 infection, including age, sex, ethnicity and contact with a SARS-CoV-2 positive person. The final regression model included adjustment for age and sex and any other variable that had a p value of <0.05 in the univariable logistic regression analyses. Odd ratios are reported with 95% confidence intervals. Statistical analysis was done using STATA v.13.

We initially planned to recruit 30 schools across the United Kingdom with the anticipation that the number of schools acquiring a testing platform will increase over the academic year. However, when schools reopened in March 2021, twice weekly lateral flow testing with an antigen test became the government recommendation. Therefore, all schools enrolled up to the end of the autumn term were included in this study. 

Ethics approval was granted by University of Cambridge Human Biology Research Ethics Committee- HRREC.202.0.39. 

## Results

Over the course of the Autumn term, 13 schools were recruited to the study and 12 of these returned data. Six schools were in South East, three in East and three in South West England. Schools were either exclusively boarding or mixed day and boarding. 697 SARS-CoV-2 rapid tests were performed between the 1 ^st^ of September 2020 and the 16th of December 2020 (end of the Autumn term). The median age of those tested was 16 years (IQR 14- 24), 44% (309/696) were male ( Table 1). 30% (208/692) of those tested were staff, 60% (416/692) were students and 10% (68/692) were household members of staff. 64% of tests were done in symptomatic infection, the majority of whom had symptoms of either fever or cough and occasionally anosmia. Other common reasons for testing included contact testing (11.9%), asymptomatic screening (14.8%) and fitness to fly (6.2%). Less common reasons (2.7%) included visiting vulnerable relatives, testing prior to hospital admission and testing for reassurance. 97% (668/692) of tests were valid, 6.7% (45/668) of which were positive for SARS-CoV-2.

**Table 1.** Clinical and demographic details of the study population. *n/N is presented when data are missing, IQR interquartile range.

	Percentage, N=697 ( *n/N)
Median age in years (IQR)	16 (14-24) (608/697)
Male	44.4 (309/696)
Designation Student Staff Household member	60.1 (416/692) 30.1 (208/692) 9.8 (68/692)
SARS-CoV-2 result Positive Negative Invalid	6.5 (45/692) 90.0 (623/692) 3.5 (24/692)
Reason for test Symptoms Contact of COVID-19 positive person Screening Travel abroad Other	64.4 (427/663) 11.9 (79/663) 14.8 (98/663) 6.2 (41/663) 2.7 (18/663)
Symptoms Cough Fever Anosmia Other	47.6 (201/422) 40.8 (172/422) 8.8 (37/422) 2.8 (12/422)

**Figure 1.**
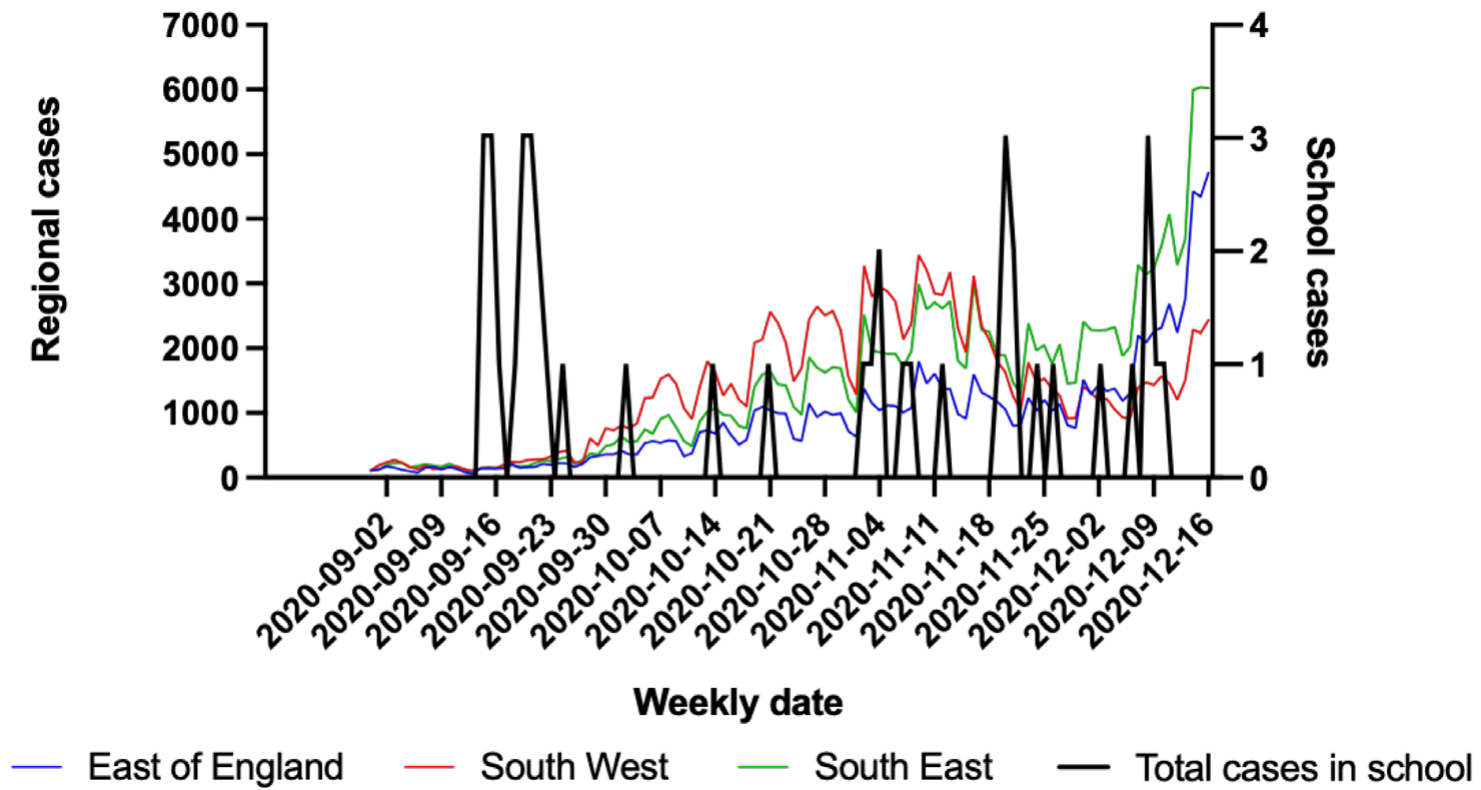
Weekly total regional COVID-19 cases numbers from East (blue), South West (red), South East (green) England on the left axis. Total weekly number of cases in schools sampled (black) on the right axis. Data on case numbers from East, South West and South East England regions were obtained from Public Health England website: https://coronavirus.data.gov.uk/details/download.

In September 2020, there was a relatively low prevalence of SARS-CoV-2 cases in the regions from which these schools were sampled and this increased over the course of the Autumn-Winter 2020 ( Figure 1). In comparison, the overall number of cases of SARS-COV-2 diagnosed in the sampled schools remained relatively low ( Figure 1) and did not increase as the second wave of the pandemic in the UK progressed. This was thought to be due to limited mixing between boarders and the local population.

Of note, a positive test for SARS-CoV-2 infection was associated with being symptomatic or being a contact of a COVID-19 case ( Table 2). No asymptomatic cases were identified amongst individuals tested for reasons such as asymptomatic screening, fitness to fly or other reasons. In the univariable logistic regression analyses, factors associated with a SARS-CoV-2 positive test result included having symptoms of anosmia [OR 7.3 (95% CI 2.9-18.5)], attending a school in the South East region [OR 4.2 (95% CI 1.8-9.7)] or South West region [OR 3.2 (95% CI 1.2-8.3)]. However, in the multivariable model adjusted for sex, age and all variables with a p value <0.05 in the univariable analyses, only anosmia maintained a significant association with a positive SARS-CoV-2 test result [OR 5.5 (95% CI 2.1-14.7, p 0.001)].

**Table 2.** Clinical and demographic factors associated with SARS-CoV-2 infection status. OR odds ratio, CI confidence interval. *n/N is presented when data are missing; - indicates where categories without any events were excluded from the analyses; ^a^ adjusted for age and sex, region and symptoms.

	Number	Risk of SARS-CoV-2 positivity	Unadjusted OR (95% CI)	P value	Adjusted OR ^a^ (95% CI)	P value
Age in years <16 ≥16	278 305	9.4 (26/278) 6.2 (19/305)	1 0.6 (0.3-1.2)	0.16		
Sex Female Male	377 290	6.4 (24/377) 7.2 (21/290)	1 1.1 (0.6-2.1)	0.66		
Designation Student Staff Household member	402 198 65	8.0 (32/402) 5.1 (10/198) 3.1 (2/65)	1 0.6 (0.3-1.3) 0.4 (0.1-1.6)	0.19 0.18		
Region East of England South East South West	264 265 139	2.7 (7/264) 10.2 (27/265) 7.9 (11/139)	1 4.2 (1.8-9.7) 3.2 (1.2-8.3)	0.001 0.02	1 2.6 (1.0-6.9) 3.1 (1.0-9.3)	0.06 0.05
Reason for test Symptoms Contact of COVID-19 positive person Screening Travel abroad Other	410 78 94 39 16	8.8 (36/410) 9.0 (7/78) 0 (0/94) 0 (0/39) 0 (0/16)	1 (0.4-2.4) - - -	0.96		
Symptoms Cough Fever Anosmia Other	193 166 36 0	5.7 (11/193) 9.0 (15/166) 30.1 (11/36) 0 (0/12)	1 1.6 (0.7-3.7) 7.3 (2.9-18.5) -	0.23 <0.001	1 1.6 (0.7-3.7) 5.5 (2.1-14.7)	0.25 0.001

Impact on school attendance

**Figure 2.**
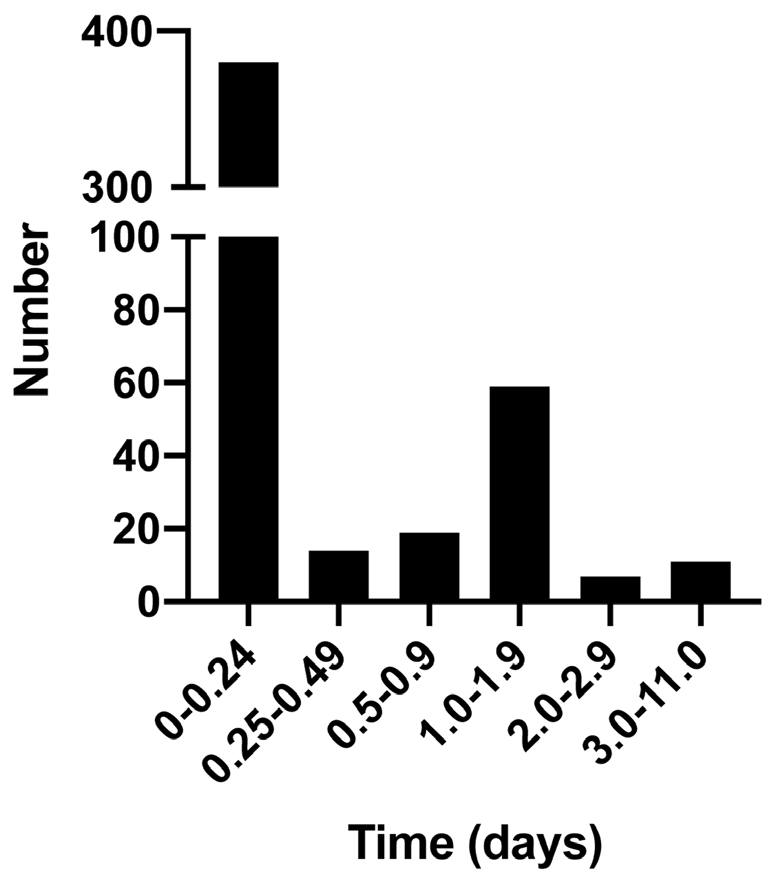
Days of absence from school whilst awaiting rapid point of care SARS-CoV-2 test result.

All infections occurred in eight out of the 12 schools. Two schools each had a single case and were able to limit transmission by identification and isolation of the infected individual. There were five outbreaks in three schools, defined as more than two cases diagnosed within two consecutive days. These outbreaks involved 16, five and four individuals in the affected schools. There were seven bubble closures and no school closures. The ability to exclude SARS-CoV-2 infection in symptomatic individuals was important in reducing school absence. The majority of individuals tested (84%) were absent from school for less than one day prior to receiving a rapid test result ( Figure 2). This compared favourably with the NHS PCR test typically done in a drive-through testing centre or a test delivered my post. It usually takes between one to three days to return a test result ( NHS coronavirus testing). Potentially, this cumulatively saves between 1,047 and 1,570 days off school (84% of all those testing negative). Participants reported that the use of an onsite rapid PCR tests helped the schools to function as fully as possible during the pandemic (Extended data 2) ^11^.

## Discussion

The proportional prevalence of SARS-CoV-2 in our study population is high at 6% as the majority of those tested had symptoms or were COVID-19 contacts. Despite this, there were no school closures and absence from school was minimal. A nationwide surveillance study of COVID-19 infection in students and staff in English schools (COVID-19 Schools Infection Survey) reported a prevalence of SARS-CoV-2 infection in secondary school students and staff of 1.48% and 1.47%, respectively, in the first half of the Autumn term from the 3 ^rd^ to the 19 ^th^ of November 2020 ^12^. The nationwide surveillance study included asymptomatic staff and students, which likely explains the difference in positivity rates between this survey population and the nationwide surveillance study. However, the incidence rates have been steadily increasing with the resultant increase in school absence and closure over the course of the Autumn term. 9-11% of children did not attend school for COVID-19 related reasons and up to 2% of state-funded schools were closed in the last week of the Autumn term ( school attendance during SARS-CoV-2 outbreak). We estimate that having a rapid POC PCR-based test in these school saved between 1,047 and 1,570 days off school compared to the National Health Service / Public Health England PCR test and made a significant difference in the running of these schools.

New guidelines from the UK government to introduce mass testing in schools is welcome, if not overdue ^13^. However, concerns have been raised about the ability of these lateral flow tests to accurately diagnose SARS-CoV-2 infection ^14^. Although the specificity of these test are high, sensitivity may be as low as 48% in asymptomatic ^8^ and 58% in symptomatic individuals ^7^ when self-administered. There is a trade-off between more expensive, highly accurate tests such as PCR tests and cheaper, less accurate, tests such as those based on lateral flow technology.

A limitation of the study is that the sample may not be representative of most schools in England in terms of being able to afford these tests, having staff capacity to implement a testing program, and the characteristics of the students attending these schools. Although a direct comparison has not been made between a POC testing platform sited on the school premises and one off-site, it is likely that the logistics required to facilitate an off-site test will add further delays to turn-around times.

We recommend that accurate, rapid POC PCR testing platforms should be widely available and utilised in school settings. We acknowledge that a barrier to implementing rapid POC PCR testing widely across all schools is cost. However, the cost to schools could be limited by government investment in these tests to support schools. We call for new research to develop cheaper but still accurate POC PCR tests. Furthermore, implementation could be managed with more expensive POC PCR tests in higher risk settings like colleges and secondary schools whilst allowing for the use of cheaper but less accurate tests in younger ages where the risk of spreading infection is lower. Reliable positive tests will prevent outbreaks and uncontrolled spread of infection within school settings. Reliable negative test results will reassure students, parents and staff and prevent disruption of schooling.

## Data availability

### Underlying data

UCL Research Data Repository: Data underpinning Point of care SARS-CoV-2 nucleic acid testing in schools improves school attendance. https://doi.org/10.5522/04/16764511 ^15^.

### Extended data

UCL Research Data Repository: Extended data 1 for Point of care SARS-CoV-2 nucleic acid testing in schools improves school attendance. https://doi.org/10.5522/04/16764535 ^10^.

UCL Research Data Repository: Extended data 2 for Point of care SARS-CoV-2 nucleic acid testing in schools improves school attendance. https://doi.org/10.5522/04/16766581 ^11^.

Data are available under the terms of the Creative Commons Zero "No rights reserved" data waiver (CC0 1.0 Public domain dedication).
