# Supplementary material for: Point of care SARS-CoV-2 nucleic acid testing in schools improves school attendance
Source: Wellcome Open Res. 2022 Sep 6;7:8. Originally published 2022 Jan 10. [Version 2] doi: 10.12688/wellcomeopenres.17213.2 (PMC9530621; doi:10.12688/wellcomeopenres.17213.2)
Supplement: Peer review history

# Peer review: Reviewer response for version 2

**DOI:** 10.21956/wellcomeopenres.20169.r52267

**Published:** 2022-10-03

**Authors:** Sarah Logan

**Affiliations:** 1 Hospital for Tropical Diseases, Division of Infection, University College London Hospitals NHS Foundation Trust, London, UK

I have reviewed the revised manuscript and tried to comment that I am happy with the edits.

Is the work clearly and accurately presented and does it cite the current literature?

Partly

If applicable, is the statistical analysis and its interpretation appropriate?

I cannot comment. A qualified statistician is required.

Are all the source data underlying the results available to ensure full reproducibility?

Yes

Is the study design appropriate and is the work technically sound?

Yes

Are the conclusions drawn adequately supported by the results?

Yes

Are sufficient details of methods and analysis provided to allow replication by others?

Yes

Reviewer Expertise:

Clinician in ID and parent of 3 children, the lived experience of school absences/ closures on the whole family and medical workforce cannot be underestimated!

I confirm that I have read this submission and believe that I have an appropriate level of expertise to confirm that it is of an acceptable scientific standard.

# Peer review: Reviewer response for version 2

**DOI:** 10.21956/wellcomeopenres.20169.r52268

**Published:** 2022-09-30

**Authors:** Sanjat Kanjilal

**Affiliations:** 1 Department of Population Medicine, Harvard Medical School and Harvard Pilgrim Healthcare Institute, Boston, MA, USA; 2 Division of Infectious Diseases, Brigham & Women's Hospital, Boston, MA, USA

I approve this submission for indexing.

Is the work clearly and accurately presented and does it cite the current literature?

Partly

If applicable, is the statistical analysis and its interpretation appropriate?

Yes

Are all the source data underlying the results available to ensure full reproducibility?

Yes

Is the study design appropriate and is the work technically sound?

Yes

Are the conclusions drawn adequately supported by the results?

Partly

Are sufficient details of methods and analysis provided to allow replication by others?

Yes

Reviewer Expertise:

NA

I confirm that I have read this submission and believe that I have an appropriate level of expertise to confirm that it is of an acceptable scientific standard.

# Peer review: Reviewer response for version 1

**DOI:** 10.21956/wellcomeopenres.19021.r49649

**Published:** 2022-04-19

**Authors:** Sanjat Kanjilal

**Affiliations:** 1 Department of Population Medicine, Harvard Medical School and Harvard Pilgrim Healthcare Institute, Boston, MA, USA; 2 Division of Infectious Diseases, Brigham & Women's Hospital, Boston, MA, USA

**Summary:**

 Collier et al performed a retrospective survey-based analysis of school-based SARS-CoV-2 testing program using a point of care PCR assay, the SAMBA II. The authors analyzed data collected from September through December 2020 when SARS-CoV-2 prevalence was 6.7% in their schools. The majority of people were symptomatic and 84% of those tested with symptoms missed less than one day of school prior to being ruled out by the rapid PCR test. Compared to the testing program for the larger community, the authors estimate that the point of care PCR testing program saved between 1,047 and 1,570 days off from school.

**Major comments:**

1. The SARS-CoV-2 Omicron variant is much more transmissible than the viral lineages that were in circulation at the time of this study in the winter of 2020. Despite a much more established testing infrastructure, the Omicron variant spread widely, suggesting that diagnostic test performance regardless of assay type significantly declined. There are many possible reasons for this but it suggests that the efficacy of any testing program based on data from prior waves is at risk for being over-estimated if that program was implemented in the current environment.
2. Generalizable conclusions are a bit challenging to draw from this study. Additional information that would be helpful for the community include:
  1. Key aspects of the testing programs, such as criteria for testing, frequency of testing and body sampling site by school (if available). Each school's definition of 'bubble' can also help readers understand how many people would be in quarantine with each positive case.
  2. Test characteristics of the SAMBA II including gene targets, limit of detection, turnaround time and literature supporting test performance.
  3. Surveillance data documenting the predominating variants in circulation at the time of the study (see #1 above).
3. Similarly, would consider broadening the discussion to more critically examine the generalizability of results. Might an alternative explanation for the success of the testing program be due to the lack of mixing in the community, and not the characteristics of their point of care PCR testing strategy? Given that up to 90% of new infections occur from an asymptomatic index case, the fact that most (perhaps all?) cases detected in this program were symptomatic suggests a highly insular environment, which again may not be the case in most other settings.
4. Regarding the following statement: "However, concerns have been raised about the ability of these lateral flow tests to accurately diagnose SARS-CoV-2 infection" Would consider citing the more recent study by Deeks in BMJ (Deeks *et al. *(2021 ^1^)).

**Minor comments:**

1. References 6 and 9 appear to be duplicated.
2. A turnaround time of 1 to 3 days is assumed for PCR testing through Public Health England. While this makes sense for 2020, is that still the case in 2022?
3. Schools were selected through a convenience sample. Is there any data to support or refute the fact that these schools are representative of schools throughout the UK and beyond?
4. With regards to the following statement: "Data collected includes test performed from the 1st of September 2020 and will be collected up till the end of the academic year in August 2021. This initial report is based on data collected in the first term of the academic year up until the 16th of December 2020.", it has been nearly 8 months since the end of August 2021, therefore a reader would reasonably expect that that data should be included in this report. Is there a reason it has not yet been included? If it cannot be added, would suggest modifying this statement to just state the study ended in December 2020.
5. In the results, it states that 697 tests were performed, however the denominators for gender, staff vs student, and household members are 696, 692, and 692, respectively. What is the reason for the discrepancy?
6. This statement could use further clarity: "No asymptomatic cases were identified amongst individuals tested for reasons such as asymptomatic screening, fitness to fly or other reasons."
  1. The way it is currently written implies there are positive tests from asymptomatic cases tested for other indications. However, my understanding based on the manuscript is that this program did not pick up any asymptomatic cases.

Is the work clearly and accurately presented and does it cite the current literature?

Partly

If applicable, is the statistical analysis and its interpretation appropriate?

Yes

Are all the source data underlying the results available to ensure full reproducibility?

Yes

Is the study design appropriate and is the work technically sound?

Yes

Are the conclusions drawn adequately supported by the results?

Partly

Are sufficient details of methods and analysis provided to allow replication by others?

Yes

Reviewer Expertise:

Clinical microbiology, infectious diseases, epidemiology, machine learning, clinical decision support

I confirm that I have read this submission and believe that I have an appropriate level of expertise to confirm that it is of an acceptable scientific standard, however I have significant reservations, as outlined above.

## References

[1] : SARS-CoV-2 antigen lateral flow tests for detecting infectious people: linked data analysis. *BMJ* .2022; 10.1136/bmj-2021-066871 10.1136/bmj-2021-066871 PMC886447535197270

# Response

**Published:** 2022-08-05

**Authors:** Dami Collier

**Affiliations:** university college london, UK

**Reviewer:** *****Collier et al performed a retrospective survey-based analysis of school-based SARS-CoV-2 testing program using a point of care PCR assay, the SAMBA II. The authors analyzed data collected from September through December 2020 when SARS-CoV-2 prevalence was 6.7% in their schools. The majority of people were symptomatic and 84% of those tested with symptoms missed less than one day of school prior to being ruled out by the rapid PCR test. Compared to the testing program for the larger community, the authors estimate that the point of care PCR testing program saved between 1,047 and 1,570 days off from school.*

*Major comments:*

*The SARS-CoV-2 Omicron variant is much more transmissible than the viral lineages that were in circulation at the time of this study in the winter of 2020. Despite a much more established testing infrastructure, the Omicron variant spread widely, suggesting that diagnostic test performance regardless of assay type significantly declined. There are many possible reasons for this but it suggests that the efficacy of any testing program based on data from prior waves is at risk for being over-estimated if that program was implemented in the current environment.*

**Author Response: **As rightly pointed out by the reviewer there are many possible reasons why the Omicron variant spread widely. However, we do not agree that a reduction in diagnostic accuracy has been a major contributor to this. The S-gene target failure (SGTF) as a result of a deletion at nt207-212 (Δ69-70) in the Spike gene was recognised relatively early in our setting. Most assays use targets other than the S-gene and although a weak or absent signal would be seen in the S-gene, a positive signal will be seen in other regions such as ORF1 and N-gene in targeted RT-PCRs.

**Reviewer:**** *Generalizable conclusions are a bit challenging to draw from this study. Additional information that would be helpful for the community include:*

*Key aspects of the testing programs, such as criteria for testing, frequency of testing and body sampling site by school (if available). Each school's definition of 'bubble' can also help readers understand how many people would be in quarantine with each positive case.*  

**Author Response: **This has now been included in the methods and references.

 “All schools utilised the SAMBA II SARS-CoV-2 testing platform, which amplifies two regions of the genome ORF1ab and nucleocapsid protein. The tests were performed on combined nose and throat swab samples by a school nurse who had received training from the manufacturer. The two regions of the genome (open reading frame 1ab [ORF1ab] and nucleocapsid protein [N]) limit of detection (LOD) of the SAMBA II SARS-CoV-2 test is 250 copies/ml ( **https://doi.org/10.1128/JCM.01262-20**).”

**Reviewer:** *Test characteristics of the SAMBA II including gene targets, limit of detection, turnaround time and literature supporting test performance.*

**Author Response: **This has now been included on the methods and references. See above *.*

**Reviewer:** *Surveillance data documenting the predominating variants in circulation at the time of the study (see #1 above).*

**Author Response: **This has now been included in the text.

 “The alpha variant was emerging as the dominant variant during this period and eventually replaced the preceding D614G wildtype variant (PHE Investigation of novel SARS-CoV-2 variant 202012/01: technical briefing 1 https://assets.publishing.service.gov.uk/government/uploads/system/uploads/attachment_data/file/959438/Technical_Briefing_VOC_SH_NJL2_SH2.pdf).”

**Reviewer:** *Similarly, would consider broadening the discussion to more critically examine the generalizability of results. Might an alternative explanation for the success of the testing program be due to the lack of mixing in the community, and not the characteristics of their point of care PCR testing strategy? Given that up to 90% of new infections occur from an asymptomatic index case, the fact that most (perhaps all?) cases detected in this program were symptomatic suggests a highly insular environment, which again may not be the case in most other settings.*

**Author Response: ** We agree and have broadened the discussion to include this.

 “A limitation of the study is that the sample may not be representative of most schools in England in terms of being able to afford these tests, having staff capacity to implement a testing program, and the characteristics of the students attending these schools. Although a direct comparison has not been made between a POC testing platform sited on the school premises and one off-site, it is likely that the logistics required to facilitate an off-site test will add further delays to turn-around times. The success of this strategy may not be solely due to the testing strategy. These populations are largely closed with limited mixing with the wider population as indicated by the low number of positives in these schools compared with the background prevalence in the geographical regions where these schools were based.”

**Reviewer:** *Regarding the following statement: "However, concerns have been raised about the ability of these lateral flow tests to accurately diagnose SARS-CoV-2 infection" Would consider citing the more recent study by Deeks in BMJ (Deeks et al. (20211)).*

**Author Response: **This reference has been cited as suggested.

 “Deeks J J, Singanayagam A, Houston H, Sitch A J, Hakki S, Dunning J *et al.* SARS-CoV-2 antigen lateral flow tests for detecting infectious people: linked data analysis BMJ 2022; 376 :e066871 doi:10.1136/bmj-2021-066871”

**Reviewer:** *References 6 and 9 appear to be duplicated.*

**Author Response: **Our apologies. This has been corrected.

 Reference 6 is:

 Collier DA, Assennato SM, Warne B, Sithole N, Sharrocks K, Ritchie A, *et al.* Point of Care Nucleic Acid Testing for SARS-CoV-2 in Hospitalized Patients: A Clinical Validation Trial and Implementation Study. Cell Rep Med. 2020;1(5):100062.

 Reference 9 is:

 Mlcochova P, Collier D, Ritchie A, Assennato SM, Hosmillo M, Goel N, *et al.* Combined Point-of-Care Nucleic Acid and Antibody Testing for SARS-CoV-2 following Emergence of D614G Spike Variant. Cell Rep Med. 2020;1(6):100099.

**Reviewer:** *A turnaround time of 1 to 3 days is assumed for PCR testing through Public Health England. While this makes sense for 2020, is that still the case in 2022?*

**Author Response: **The turnaround time for PCR testing has varied depending on the stage of the pandemic with longer turnaround times during periods of higher incidence which the resulting pressures of laboratories. The turnaround time quoted (supported by the reference) was the turnaround time at the time the study was conducted.

**Reviewer:** *Schools were selected through a convenience sample. Is there any data to support or refute the fact that these schools are representative of schools throughout the UK and beyond?*

**Author Response: **We stated in both the methods and references, these schools are fee-paying schools and so not representative of schools throughout the UK.

 “A limitation of the study is that the sample may not be representative of most schools in England in terms of being able to afford these tests, having staff capacity to implement a testing program, and the characteristics of the students attending these schools.”

**Reviewer:**** *With regards to the following statement: "Data collected includes test performed from the 1st of September 2020 and will be collected up till the end of the academic year in August 2021. This initial report is based on data collected in the first term of the academic year up until the 16th of December 2020.", it has been nearly 8 months since the end of August 2021, therefore a reader would reasonably expect that that data should be included in this report. Is there a reason it has not yet been included? If it cannot be added, would suggest modifying this statement to just state the study ended in December 2020.*

**Author response: **We stated in the methods the study was stopped after the initial phase. 

*“*We initially planned to recruit 30 schools across the United Kingdom with the anticipation that the number of schools acquiring a testing platform will increase over the academic year. However, when schools reopened in March 2021, twice weekly lateral flow testing with an antigen test became the government recommendation. Therefore, all schools enrolled up to the end of the autumn term were included in this study.”

   

**Reviewer:**** *In the results, it states that 697 tests were performed, however the denominators for gender, staff vs student, and household members are 696, 692, and 692, respectively. What is the reason for the discrepancy?*

**Author response: **697 tests were done, however when data are missing, we present the denominator as non-missing data. Please refer to table 1. *n/N is presented when data are missing.

**Reviewer:** *This statement could use further clarity: "No asymptomatic cases were identified amongst individuals tested for reasons such as asymptomatic screening, fitness to fly or other reasons."*

*The way it is currently written implies there are positive tests from asymptomatic cases tested for other indications. However, my understanding based on the manuscript is that this program did not pick up any asymptomatic cases.*

**Author response: **This sentence has been clarified and now reads.

*“Seven asymptomatic cases were identified who were contacts of known SARS-CoV-2 positive cases”*

# Peer review: Reviewer response for version 1

**DOI:** 10.21956/wellcomeopenres.19021.r48795

**Published:** 2022-02-23

**Authors:** Sarah Logan

**Affiliations:** 1 Hospital for Tropical Diseases, Division of Infection, University College London Hospitals NHS Foundation Trust, London, UK

For understandable reasons there is quite a delay in getting this to publication and the landscape has changed. Nonetheless the number of school days saved by deployment of a rapid POC PCR based test is sizeable and the impact of a sensitive diagnostic test can have on enabling school attendance is a key point to learn for future.

 More can be made in the discussion about the generalizability of this sort of approach in pandemics. Rapid roll out of diagnostics and evaluation of their impact over and above their PPV and NPV was a missing part of the approach to this pandemic. Therefore I do think that there is value in publication at this stage if the emphasis of the manuscript can be more on what can we learn and do better for next time.

Is the work clearly and accurately presented and does it cite the current literature?

Partly

If applicable, is the statistical analysis and its interpretation appropriate?

I cannot comment. A qualified statistician is required.

Are all the source data underlying the results available to ensure full reproducibility?

Yes

Is the study design appropriate and is the work technically sound?

Yes

Are the conclusions drawn adequately supported by the results?

Yes

Are sufficient details of methods and analysis provided to allow replication by others?

Yes

Reviewer Expertise:

Clinician in ID and parent of 3 children, the lived experience of school absences/ closures on the whole family and medical workforce cannot be underestimated!

I confirm that I have read this submission and believe that I have an appropriate level of expertise to confirm that it is of an acceptable scientific standard, however I have significant reservations, as outlined above.

# Response

**Published:** 2022-08-05

**Authors:** Dami Collier

**Affiliations:** university college london, UK

**Reviewer:** *For understandable reasons there is quite a delay in getting this to publication and the landscape has changed. Nonetheless the number of school days saved by deployment of a rapid POC PCR based test is sizeable and the impact of a sensitive diagnostic test can have on enabling school attendance is a key point to learn for future.*

**Author Response:** Many thanks to the reviewer for this comment. This is the main thrust of this article.

**Reviewer:** *More can be made in the discussion about the generalizability of this sort of approach in pandemics.*

**Author Response: **This has now been addressed in the discussion.

**Reviewer:** *“Rapid diagnostic tests have a role to play in emerging infectious diseases and as has been demonstrated in this study in schools, it can be rolled-out to assist in managing contacts, allowing prompt isolation of new cases and preventing absenteeism.”*

*Rapid roll out of diagnostics and evaluation of their impact over and above their PPV and NPV was a missing part of the approach to this pandemic. Therefore I do think that there is value in publication at this stage if the emphasis of the manuscript can be more on what can we learn and do better for next time.*

**Author Response: **We wholeheartedly agree with this comment and indeed the landscape has changed since this study was conducted. We do believe that what this study shows is a workable option for managing COVID transmission in a closed setting. Its wider adoption may be limited by cost but early in an emerging epidemic when there isn’t widespread community transmission, highly accurate, rapid tests such as these certainly have a role. In light of the changed landscape of the pandemic we have removed the recommendation that rapid POC PCR testing platforms should be widely available and utilised in school settings from the discussion.

 “There is a trade-off between more expensive, highly accurate tests such as PCR tests and cheaper, less accurate, tests such as those based on lateral flow technology.”

 And highlight, where they could potentially have worked, had these technologies been adopted in the Autumn of 2020.

 “We call for new research to develop cheaper but still accurate POC PCR tests. Furthermore, implementation could be managed with more expensive POC PCR tests in higher risk settings and   like colleges and secondary schools whilst allowing for the use of cheaper but less accurate tests in younger ages where the risk of spreading infection is lower.”
